# Doctors’ views of disulfiram and their response to relapse in alcohol-dependent patients, Free State, 2009

**DOI:** 10.4102/phcfm.v8i1.1053

**Published:** 2016-06-17

**Authors:** Paulina M. van Zyl

**Affiliations:** 1 Department of Pharmacology, Faculty of Health Sciences, University of the Free State, South Africa

## Abstract

**Background:**

Disulfiram is the oldest and best known drug to prevent relapse after detoxification from alcohol. Effective use of the drug is dependent on stringent monitoring and high levels of external motivation. Doctors’ perceptions about the drug have not been investigated extensively.

**Aim:**

We investigated the perceptions and practices of doctors involved in relapse prevention in alcoholics with regard to disulfiram and their response to relapse.

**Setting:**

The study population consisted of 60 doctors from the Free State Province, involved in the follow-up of alcoholics across various work settings.

**Methods:**

A cross-sectional descriptive study design was used, and data collection involved the use of a questionnaire and semi-structured interviews. Quantitative results are presented in figures and percentages to provide a background for the qualitative findings that are clustered in themes.

**Results:**

A quarter of participants did not prescribe disulfiram, another quarter prescribed disulfiram routinely after detoxification, and half of them prescribed it for selected cases only. Subject to affordability, selection of disulfiram was mainly determined by the perceived level of the patient’s motivation. External motivation sometimes took the form of threats of bodily harm or death caused by drinking. Some participants regarded relapse as confirmation of poor motivation and even a valid reason for terminating the doctor-patient relationship.

**Conclusion:**

Doctors perceive disulfiram as a psychological tool to induce motivation through creating fear of drinking. Failure and success are perceived as related to the level of motivation. These perceptions could be unfair as biological factors in inter-patient variability in response are ignored.

## Introduction

Relapse after alcohol detoxification is common, despite expensive multidisciplinary intervention. Disulfiram (Antabuse^®^) is the oldest and best known drug prescribed to prevent relapse in alcohol dependence.^1,2^ The primary pharmacological mechanism of disulfiram is the irreversible inhibition of aldehyde dehydrogenase that causes the accumulation of acetaldehyde when disulfiram is taken concurrently with alcohol. The unpleasant effect that follows provides a psychological intervention intended to make alcohol intake an experience to be avoided.^3^ Disulfiram is also known as a potent inhibitor of dopamine β*-*hydroxylase (DBH), the enzyme that mediates the conversion of dopamine to noradrenaline.^4^ The resultant accumulation of dopamine is thought to counteract dopamine deficiency that underlies craving, hence, disulfiram is also an anti-craving drug. The latter effect is strikingly demonstrated in cases of codependence on alcohol and cocaine.^4^

Disulfiram remains an important cornerstone of pharmacotherapy for relapse prevention in alcoholism because of its comparative effectiveness when compared to naltrexone and acamprosate.^1,2^ Yet, despite the continued and widespread use of disulfiram for this indication, its effectiveness in the treatment of alcoholism has been questioned for several decades,^5^ mainly because of the requirement of stringent external monitoring and motivation to ensure the success of the intervention.

Earlier studies on the effectiveness of disulfiram in the treatment of alcoholism provided inconsistent results, but it was the so-called ‘veteran study’, published in 1986,^6^ that gave the drug the reputation of a placebo. This study was the largest placebo-controlled investigation up to that time and involved 605 war veterans, randomised to receive disulfiram daily at 250 mg, at 1 mg or placebo. Disulfiram in this case showed no advantage compared to placebo in terms of abstinence, time to first drink, employment, or social stability.

Chick et al.^7^ provided strong counter-evidence, showing advantage for disulfiram above placebo when administered through directly observed administration. Its application in employee assistance programmes (EAPs) and probation practices resulted in greatly improved work attendance and decline in alcohol-related crime, as well as improved outcomes in repetitive defaulters. Relative success was, however, dependent on stringent monitoring and motivation provided by the probation personnel and threats of discharge.

Hughes and Cook^8^ concluded that placebo-controlled studies failed to prove benefit in preventing relapse in alcohol-dependent persons. Yet, the 2012 review by Krampe and Ehrenreich^9^ confirmed a turn of the tide with all clinical studies where disulfiram was administered with adequate supervision, published from 2000 to 2008, reporting disulfiram to be effective. These authors concluded that successful treatment with disulfiram was dependent on the psychological aspects surrounding the drug and was independent of the dose.

Finally, the debate on the efficacy of disulfiram was resolved by the process of meta-analysis. Jørgensen et al.^10^ published the results of a decisive meta-analysis involving 11 studies and more than 1500 cases in favour of disulfiram. Although the included studies differed in terms of inclusion criteria and recruitment practices, the authors could demonstrate an advantage of disulfiram over placebo, no treatment, and other treatments, with regard to duration of abstinence and number of drinking days over periods ranging from 2 to 12 months. Disulfiram’s comparative effectiveness is therefore highly context-sensitive; it is only better than placebo when used in strictly monitored conditions with provision for high levels of external motivation. A later meta-analysis of 22 studies by Skinner et al.^11^ concluded that disulfiram was only superior to placebo in open-labelled studies, meaning that it is not possible to exclude a psychological effect.

In practice, the psychological aspects involved in disulfiram treatment pose unique challenges to the doctor-patient relationship, which in turn create the platform for all clinical interactions involved in diagnosis and therapy, including the basis of trust and motivation.^12^

Very limited literature exists on doctors’ views on disulfiram. Mark et al.^13^ investigated prescribers’ views regarding the efficacy and safety of disulfiram in 1388 physicians specialising in addiction medicine. Forty-nine percent of their participants rated the efficacy of disulfiram as ‘good/very good/excellent’, and 51% rated the safety of disulfiram as ‘good/very good/excellent’. In comparison, the 14% of the population rating the efficacy of disulfiram as ‘poor’ was considerably higher than the corresponding 7% rating for naltrexone. Likewise, the 11% rating disulfiram’s safety as ‘poor’ was considerably higher than the less than 1% rating naltrexone’s safety as ‘poor’.

In a survey among 1361 post-graduate trainee medical specialists, Roche et al.^14^ found that only 16% of the participants considered the evidence in support of the use of disulfiram as ‘strong’. Ogborne et al.^15^ reported that first-line staff in treatment programmes in Ontario rated the contribution of pharmacotherapy in the treatment of dependence in general as ‘low’, including the use of disulfiram in alcohol dependence.

Exploration of this neglected interface is needed for greater understanding of the problems facing prospective prescribers of disulfiram to improve outcome through the most appropriate use of the drug.

We argue that the extraordinary monitoring needs that accompany the use of disulfiram will necessarily influence the doctors’ attitudes and the doctor-patient relationship, which in turn might play an important role in maintaining patients in therapy.

### Aim and objectives

The aim of the study was to explore the attitudes, perceptions, and experiences of doctors involved in the treatment of alcohol dependence towards disulfiram and their responses to relapse.

### Significance of the study

The study contributes to the limited body of literature regarding the viewpoints of doctors towards disulfiram, and to our knowledge, is the first to explore the responses of doctors to relapse. We believe that this may play a significant role in the use of disulfiram in particular and the effectiveness of relapse prevention in general.

## Research methods and design

### Study design

A cross-sectional descriptive study with both qualitative and quantitative elements was performed.

### Setting

The study was performed in private practices of general doctors or psychiatrists and state hospital settings in the Free State Province, South Africa in 2009.

### Sample population and sampling strategy

The sample population represented doctors involved in the management of patients after detoxification from alcohol. The data presented here are extracted from a larger study^16^ involving 121 doctors and therapists in public and private work settings who could reasonably be expected to be confronted by the problem of alcohol dependence. One hundred and seven (107) of these participants were doctors, and included 77 private general practitioners, 11 private psychiatrists, 17 medical officers or consultants at state hospitals, and two general practitioners working at treatment centres.

#### Sample size

This article considers data generated through questionnaires and semi-structured face-to-face interviews conducted with 60 of the doctors who indicated that they were involved in the follow-up of alcoholic patients after detoxification.

#### Sample selection

The initial selection of doctors in private general practice and hospitals was based on randomisation tables, while all available private psychiatrists and treatment centre practitioners were included.

### Data collection

Data were collected by means of a questionnaire and a semi-structured interview. The researcher who performed the interviews has former collegial relationships with two of the participants, another eight were known individuals, while the remaining participants were strangers. Participants were ensured that their anonymity would be maintained and encouraged to express themselves freely. All interviews were recorded and transcribed. The questionnaire contained questions about participants’ preferred pharmacotherapy interventions for alcohol-relapse prevention in a typical case according to three categories: ‘standard’ (prescribed to all cases), ‘for selected cases’ (under certain conditions), and ‘do not prescribe’. Depending on their answer, participants were asked to comment on why they would not prescribe disulfiram or in which circumstances they would prescribe it.

In the semi-structured interviews, participants were asked for their views on the role of pharmacotherapy and their response when a patient relapses. Spontaneous remarks about disulfiram were included in the dataset.

### Methodological errors

Participants had varying levels of exposure to alcohol-dependent persons, ranging from occasional exposure (less than once per month) for some general practitioners to daily exposure in treatment centres. It was not possible to completely separate participants’ perceptions of relapsing patients from their perceptions with regard to alcoholics in general.

### Data analysis

The quantitative software programme NVivo version 8^17^ was used to facilitate content and thematic analysis of the transcriptions of the recorded interviews. Qualitative findings were collated, clustering the responses of participants according to themes, to construct an overview of participants’ views on the use of disulfiram.

A selection of the quantitative data is used to sketch a background for the findings which are structured according to the emerging themes. Quotations are marked with a shortened version of the original file numbers; translations from Afrikaans are indicated by *t* added to the code.

### Ethical considerations

Approval for the study was granted by the Ethics Committee of the Faculty of Health Sciences, University of the Free State. Informed consent from participants, institutional managements, and the Free State Department of Health were obtained, where applicable.

## Results

Of the 60 participants in the selected sample, 15 (25.0%) never prescribed disulfiram, whereas 45 (75.0%) did. Of these, 13 (21.6%; *n* = 60) routinely prescribed disulfiram and 32 (53.3%; *n* = 60) prescribed it for selected cases only. Nine participants claimed that they had no access to disulfiram (Table 1). The researcher divided the material into the following main themes: (1) grounds for prescribing disulfiram therapy or for not prescribing the drug (a preconceived theme); (2) the perceived relationship between disulfiram and motivation (an emergent theme); (3) perceptions regarding the efficacy of disulfiram (an emergent theme); and (4) the doctors’ responses to relapse.

**TABLE 1:** Use of disulfiram in the different treatment settings.

Variables	General practition-ers (*n* = 41) *n* (%)	Private psychiatr-ists (*n* = 10) *n* (%)	State hospital representa-tives (*n* = 7) *n* (%)	Treatment centre representatives (*n* = 2) *n* (%)
Standard	10 (24.3)	1 (10.0)	0	2 (100.0)
Selected patients	22 (53.7)	5 (50.0)	5 (71.4)	0
Do not use	9 (22.0)	4 (40.0)	2 (28.6)	0

*Source*: Van Zyl^16^

### Reasons for prescribing disulfiram or for not prescribing disulfiram

The prescribers’ judgment of the socio-economic situation of the patient and patient motivation were the two major determinants for disulfiram prescription (Table 2). Disulfiram is relatively expensive and, according to participants, most medical aid schemes do not cover the cost of this drug.

**TABLE 2:** Reasons for avoiding disulfiram or for prescribing disulfiram.

Variables	Participant(s)
**Reasons for avoiding disulfiram**	
Ineffective	N03; N16; N38; N43
Risky/dangerous	E28; N40; S24; S31
Unavailable	N05; N07; N13; S08; S11; S28
Unaffordable	E28; E29; S41
Lack of follow-up facility	S07
Unspecified	N32; S34
**Prerequisites and indications for selecting disulfiram**	
Patient motivation and cooperation	E13; N17; N38; N39; S01; S02; S03; S36
On request by patient, family or employer	E05; E20; S03; S09; S10; S26; S30; S37
If the patient can afford it	E08; E20; E31; E29; N05; N16; N34; S32; S39
Previous relapses	S03
Patient going for treatment in an institution or is part of an EAP programme	E28; N26; S14
Stubborn drinkers with limited insight	E03
Patients needing help with self-control	E05
Patient with external locus of control	S05
Unspecified	N01; N20; N36; N37; S19; S26

*Source*: Van Zyl^16^

EAP, employee assistance programme.

Some participants considered disulfiram appropriate for ‘well motivated’ patients committed to participate in their treatment; by contrast, others reserved it for patients with a history of relapse whom they described as ‘needing help with self-control’ or ‘stubborn drinkers with limited insight’.

Certain participants adopted a compliant role, prescribing disulfiram if asked to do so by the patient or family or treatment centre, even though they themselves were unconvinced of the merit of the drug. One prescriber, for example, explained:

‘Initially it helps; I do not believe in it much, but for those who are afraid, I will give it.’ (S37*t*)

A single participant offered lack of monitoring capacity as the reason for not prescribing disulfiram routinely. Another thought that it was futile, because of the lack of patient compliance:

‘I do not think it works well. People do not drink the stuff. They throw it away.’ (N6*t*)

Further reasons given by prescribers for not prescribing the drug were that the drug was ‘ineffective’ or ‘risky’ and definitely so if administered without the patient’s knowledge:

‘It is dangerous to slip into someone’s porridge.’ (E28)

### Disulfiram and motivation

Nearly all participants supported the notion that motivation was the ultimate factor determining the success of the treatment, as reflected in the following statements:

‘It must be a motivated patient to get results.’ (N18*t*)

‘The role of pharmacotherapy is minimal. It is actually the patients that must be motivated to stop it [*drinking*].’ (N19*t*)

Most of the participants, however, were pessimistic to sceptical about the chances of a successful intervention. The few who expressed optimistic expectations related their advice for effective motivation through reminders of the dire consequences of drinking while on disulfiram:

‘You explain to him in graphic detail what will happen to him if he drinks…, because fear is the mother of morality, and you create fear of consequences.’ (E28)

‘[*I tell them*] the previous guy that I saw that went on like this, has died…’ (E02*t*)

### Doctors’ responses to relapse

Doctors’ answers to the question of what happens when a patient relapses ranged from acceptance and patience to frustration and even anger.

Most participants indicated that they remained committed to a case despite relapse, and maintained hope and optimism in the face of failure, as reflected by the following comments:

‘The fact that he failed, is not a reason not to try again.’ (E13*t*)

‘You always have a little bit of hope that the next time it will work.’ (S34*t*)

Some doctors expressed kindness and used a gentle approach:

‘I am soft on them. I understand their problems. When he relapses, we start all over again.’ (N38*t*)

Others expressed anger and were tough on their patients:

‘I am rather aggressive with them. I tell them they are wasting their own money and they must go back for treatment.’ (E05*t*)

‘When he misses an appointment, I phone his employer, I check on him. I hurt him where it matters.’ (S15*t*)

‘One does become rough. You threaten them, but you help when they ask [*for help*].’ (N37*t*)

Certain participants saw the relapsed patient as a challenge:

‘I don’t give up on people. I will help him over and over until it works.’ (S29*t*)

‘We will do our best until they die.’ (N03)

In general, the doctors were not neutral about relapse. Some related how relapse changes their attitude, approach, and behaviour towards a patient, and evoked irritation and threatening behaviour:

‘No, you do not cut off, but one is stricter with such patients.’ (E17*t*)

‘I often say: it’s the last time I will help you, but if they do come back, I will help them again.’ (E03*t*)

‘I help every time. I have one such patient; I am irritated every time I see him. He wanted to report me to the Medical Council, but whenever he returns, I help again.’ (E04*t*)

Some expressed despondency and admitted that they saw their own motivation decline:

‘I am inclined if someone comes back for the second or third time to be less motivated.’ (S02*t*).

‘I know one gets despondent, you do not succeed, the compliance is not there. You still cannot give up trying.’ (N22*t*).

‘The family is often as despondent as the doctor. We only give up because he is relapsing again and again.’ (E15*t*)

‘Let me tell you, 60% of those guys relapse despite anything that you do for them, that is unfortunately so and that is what I see.’ (E02*t*)

Several participants displayed a sense of being torn between duty as a physician and frustration at the lack of success:

‘I become cross with them. I tell them this far and no further. I give and I get nothing in return. So they must tell me whether they want to continue… If I think he will rehabilitate, I will try again. …’ (N30*t*)

Some doctors admitted that they would give up on a case or even terminate the doctor-patient relationship, justified by the perception of the patient’s lack of motivation:

‘… and if that guy, despite the threats and me going through the whole story, still continues, then I leave him.’ (E02*t*)

‘I tell the guy he should not bother me. I got up in the night for him… He is an adult and [*he*] knows the consequences of his drinking.’ (N17*t*)

‘If you don’t use the chance we are giving you now, we will not help you further.’ (N31*t*)

One participant described a sequence of initial vigour and enthusiasm, followed by intensification of the doctor’s efforts, then despondency and, finally, abandonment:

‘I recently had a patient who drank at work. [He was] threatened that he is going to lose his job and I sat with that guy week in and week out… I phoned his workplace. I later got one of his colleagues [involved] and I still manage his Antabuse. He turns up for three mornings and then he is on holiday, and then he is still not back and then… then you give up. I do not believe in giving up, it is just [that] your hands are tied.’ (E06*t*)

The occurrence of relapse, despite directly observed administration of disulfiram, caused at least one doctor not to believe in the drug:

‘Antabuse, personally I do not know how effective it is, because those that you prescribe, they don’t drink it. I am at the point where I personally dish out the pills and phone the guy every day at his work to come and fetch his pills. Even that didn’t work. So does Antabuse work for me? No.’ (E06*t*)

The same doctor reported on other cases:

‘…they drank through the Antabuse… they are still not motivated….’ (E06*t*)

Finally, many participants concluded that it was often impossible to determine the outcome of treatment with disulfiram, as they lost most cases in follow-up. In other words, patients often decided to terminate the doctor-patient relationship.

Table 1 reflects the standard practice with regard to the use of disulfiram in different treatment settings. Almost a quarter of the participants indicated that they did not prescribe disulfiram at all, despite the fact that they supported patients in alcohol-relapse prevention.

Table 3 summarises the views of participants regarding the interpretation of relapse despite the use of disulfiram. Text in quotations marks represents the participant’s verbatim response; text not in quotations marks represents a summary of several participants’ responses which had the same underlying theme.

**TABLE 3:** Summary of participants’ views regarding relapse.

Themes	Views
Always helps	Against cut-off point or black-listing (N07; N15; N46; S10; S11; S18; S19; S31; S32). Stay committed due to ethical obligations (S14). Accept relapse as part of a chronic disease (N02; N27; N45; S07; S42). ‘Always maintain hope that this time it will be successful’ (S34). ‘…even if you do it only for the family, yet [I] do not have hope that they will become better’ (S04). ‘You often feel that you should refuse further treatment, but ultimately you cannot refuse’ (N16). ‘We will talk seriously, but [I] will continue to see them’ (E08*t*). ‘We take the patients back, yet we are sceptical’ (S12).
Reassess and follow individualised approach	Relapses are readmitted for detox, not sent for rehab again, we support him through the crisis (S05; S06). ‘Patients with severe personality dysfunction relapse early: shorter admission for detox, refer to professional support’ (S05).
Refer	(E20; E31; N13; N18; N26; N36; N40; S25; S26; S28; S30).
Conditional help	The patient has to demonstrate a ‘willingness to be helped’ (E13; N18; N33; N35; S13). Set boundaries and certain terms (S33; S09). Further help depends on availability of funding (N01; N19). Set criteria or judge whether patient should get another chance (S12*t*; S13).
Aggression and threats	‘“Aggressive” or “angry” or “stricter” approach’ (E05; E28; N30). Motivate patient, involve family and employer (E18). ‘Threaten patient with the physical consequences and death; if he does not respond, terminate involvement in addiction specific treatment’ (E02). Doctor often threatens to withdraw, but always helps again (E03; N37). ‘Scold them, do not feel sorry, but may adapt pharmacotherapy’ (S44).
Giving up	Some people do not want to be helped (N27; S03; S41). ‘After the third time in a short time span, you know it will not help’ (N39). ‘Refuse further treatment’ (N17). ‘Manage the case by yourself at first, refer to treatment centre the second time and for long-term admission the third time; give up thereafter’ (N34). ‘The first six months…you would allow a third relapse at the most. Then he must take care of himself, because he is irresponsible’ (S09*t*).
Doctor becomes less motivated	‘…doctors often feel that they have to cure the patient…If the responsibility is not mine, I do not have to feel guilty if he relapses again’ (PS07*t*).
Doctor should work harder at motivation	‘[The] patient should not be penalized, the doctor shares the guilt for the failure and should work harder on support’ (S08). ‘I will check up more intensely, phone him up if he does not attend AA meetings: hurts him where it matters’ (S15). ‘… the doctor-patient relationship… can cause the patient to relapse’ (S29).

*Source*: van Zyl^16^

## Discussion

The present study reveals that in order to create the supervised, strictly monitored environment that is essential for optimal intervention with disulfiram, doctors may apply vigorous, threatening, even invasive tactics. Chick et al.^7^ pointed out that patients with an external locus of control, who are more compliant with an authoritarian relationship are more likely to respond successfully to disulfiram. It is conceivable that psychological features of the patient determining the patient’s response to the doctor’s approach underlie the frequent reports of patients not returning for follow-up. Friedman et al.^18^ provided an extensive list of strategies for primary health care physicians to deal with alcoholic patients, specifically emphasising that the patient should be made aware at the commencement of treatment that the doctor-patient relationship is not dependent on abstinence.

The finding that the selection of disulfiram for alcohol-relapse prevention in favourable socio-economic circumstances is mainly determined by the perceived level of the patient’s motivation, conforms to current recommendations regarding the use of the drug.^19^ In fact, it is generally accepted that the pharmacological effect of disulfiram is subject to its psychological role. The doctor induces external motivation through threats, and disulfiram is used as a tool to enhance the verbal threat of unpleasant physical consequences of drinking while on disulfiram. Our results show that some doctors will use the threat of grievous bodily harm, if not the threat of death itself, in their effort to provide external motivation.

The present study found that doctors regard the degree of response to disulfiram in the treatment of alcohol dependence as a measure of the patient’s level of motivation, confirming the presence or lack of commitment from the patient, and as such, even as a valid reason for termination of the doctor-patient relationship. Unfortunately, the study did not determine the typical duration of such relationships.

The current study shows how the high esteem that doctors hold for motivation plays out in criteria for selection and evaluation of outcome. The study confirms that ambiguity exists around who should get disulfiram: the ‘well motivated’ or the repeat defaulter’? Current practice guidelines are that disulfiram is indicated for well motivated patients.^19^ Ironically, addictive processes target the reward area in the brain that is responsible for motivation.^20^

In line with some of the observations reported in the study, compliance is an important prerequisite, but does not ensure success in all cases. Johnsen and Mørland,^21^ for instance, could not show any advantage for disulfiram implants, probably because of poor bio-availability. Brewer et al.^3^ argued that failure on disulfiram may occur because of inadequate dosing. Others argued that disulfiram is only effective once the patient actually experiences a disulfiram reaction.^22^

The pharmacological aspects of disulfiram appear to be widely ignored in the evaluation of outcome of the intervention in the current study population. The idea persists that the effect of disulfiram on alcohol ingestion is solely because of its psychological impact.

Studies reporting the effective use of disulfiram in cases of combined alcohol and cocaine dependence support the presence of a clinically useful anti-craving effect. Weinshenker et al.^23^ demonstrated a decreased alcohol preference in male (but not female) DBH knockout mice, and Mutschler et al.^24^ linked polymorphism of DBH to a variation in the sensitivity to disulfiram in alcoholics. The contribution of the anti-craving effect to the efficacy of the drug in alcohol-relapse prevention has not been investigated widely.

The current interpretation of the response to disulfiram may reflect an underestimation of the heterogeneity of the alcohol-dependent population, and how this manifests in inter-individual expression of drug response.

It is possible that the response of participants to relapse may be a reflection of their perception of alcoholic patients in general, intermingled with accompanying frustration. No literature reports could be found regarding physicians’ responses to relapse, and very little literature exists on how doctors perceive alcoholics. Mignon^25^ interviewed 26 physicians on their perceptions of alcoholic patients. Three quarters of interviewees did not regard alcoholism as a disease, and alcoholic patients were described as ‘unpleasant’ or ‘untrustworthy’. Though this was not the primary focus of our study, some participants in our study revealed that they believed that relapsing patients ‘did not want to be helped’ or are ‘irresponsible’.

### Study limitations

The study is limited by the fact that responses are based on recall of the participants and not on actual real time events.

### Recommendations

The decision whether or not to prescribe disulfiram should include an assessment of the capacity of the therapeutic environment to provide external motivation. This includes the dynamics of the doctor-patient relationship.

Because addicted persons suffer from compromised motivation as well as the fact that positive results for disulfiram are seen in settings with high levels of coercion, the term ‘well motivated’ should be interpreted as referring to those for whom a strong sense of motivation can be created. The purpose of such external motivation should firstly be to ensure compliance with the dosing schedule.

Further research on the reasons for the failure of disulfiram in prevention of relapse in alcohol dependence is needed to improve the prediction of outcome, and to provide guidance on the appropriate selection of individuals for disulfiram therapy, the interpretation of relapse on disulfiram, and consequent intervention.

On the other hand, the counselling skills of the doctor might be a factor in the perception of the value of disulfiram. This too needs to be explored in further studies.

## Conclusion

Doctors perceive disulfiram as a psychological tool to induce motivation through creating fear of drinking. Failure and success are perceived as related to the level of motivation. These perceptions could be unfair as biological factors in inter-patient variability in response are ignored. Doctors’ views on the effectiveness and safety of the drug and the necessity of providing stringent even intrusive motivation and monitoring may discourage some practitioners from becoming involved in such intervention.
